# Predominance of Canine Parainfluenza Virus and *Mycoplasma* in Canine Infectious Respiratory Disease Complex in Dogs

**DOI:** 10.3390/pathogens12111356

**Published:** 2023-11-15

**Authors:** Aurelle Yondo, Allen A. Kalantari, Ingrid Fernandez-Marrero, Amy McKinney, Hemant K. Naikare, Binu T. Velayudhan

**Affiliations:** 1 Athens Veterinary Diagnostic Laboratory, College of Veterinary Medicine, University of Georgia, Athens, GA 30602, USA; 2 Tifton Veterinary Diagnostic and Investigational Laboratory, College of Veterinary Medicine, University of Georgia, Tifton, GA 31793, USAnaikare@uga.edu (H.K.N.)

**Keywords:** canine, respiratory, virus, disease, diagnostics

## Abstract

Canine infectious respiratory disease complex (CIRDC) is caused by different viruses and bacteria. Viruses associated with CIRDC include canine adenovirus type 2 (CAV-2), canine distemper virus (CDV), canine influenza virus (CIV), canine herpesvirus type 1 (CHV-1), canine respiratory coronavirus (CRCoV), and canine parainfluenza virus (CPIV). Bacteria associated with CIRDC include *Bordetella bronchiseptica*, *Streptococcus equi* subspecies *zooepidemicus* (*S. zooepidemicus*), and *Mycoplasma* spp. The present study examined the prevalence of CIRDC pathogens in specimens received by a Veterinary Diagnostic Laboratory in Georgia, USA., from 2018 to 2022. Out of 459 cases, viral agents were detected in 34% of cases and bacterial agents were detected in 58% of cases. A single pathogen was detected in 31% of cases, while two or more pathogens were identified in 24% of cases. The percentages of viral agents identified were CAV-2 (4%), CDV (3%), CPIV (16%), CRCoV (7%), and CIV (2%). The percentages of bacterial agents were *B. bronchiseptica* (10%), *Mycoplasma canis* (24%), *Mycoplasma cynos* (21%), and *S. zooepidemicus* (2%). Over the five-year period, the positive cases ranged from 2–4% for CAV-2, 1–7% for CDV, 1–4% for CHV-1, 9–22% for CPIV, 4–13% for CRCoV, and 1–4% for CIV. Overall, the most prevalent pathogens associated with CIRDC were CPIV, *M. canis*, and *M. cynos*.

## 1. Introduction

Considered among the most common respiratory conditions in veterinary practice worldwide, canine infectious respiratory disease complex (CIRDC) is a multifactorial syndrome characterized by an acute onset of respiratory signs including nasal, and ocular discharge, coughing, sneezing, and fever [1]. The syndrome, also known as kennel cough, is highly contagious and the pathogens associated with this disease complex are transmitted by direct dog-to-dog contact and through airborne transmission via respiratory secretions. The clinical signs are mostly self-limiting and affected dogs may recover within days or weeks. In certain cases, the infected dogs may not show any overt clinical signs at all, and can act as asymptomatic carriers of one or more pathogens associated with the CIRDC [2]. These asymptomatic and clinically healthy dogs can act as a reservoir of CIRDC pathogens and play a role in the transmission of the disease to susceptible dogs, leading to increased morbidity [3]. However, the prevalence of such asymptomatic carriage of CIRDC pathogens is low in clinically healthy dogs [2]. On the contrary, there is a possibility that some cases may evolve into a severe form of the disease with pulmonary tissue involvement leading to pneumonia but rarely result in death [1,4].

Several viral and bacterial agents are involved in causing CIRDC in the respiratory tract, and these pathogens can sequentially or synergistically act to result in a particular clinical picture. Dogs of all ages could be infected by these respiratory pathogens, but puppies have been previously shown to be the most affected [5,6]. The most vulnerable population to CIRDC are younger dogs housed in densely populated environments, such as animal shelters and breeding kennels [7,8,9]. Co-infection is frequent, and previous studies have shown that it may result in a complicated form of the disease including pneumonia [6,7].

The principal CIRDC causative organisms are *Bordetella bronchiseptica* [10], canine adenovirus type 2 (CAV-2) [11], canine parainfluenza virus (CPIV) [12], canine herpesvirus type 1 (CHV-1) [13], and canine distemper virus (CDV) [9]. Over time, additional agents have been found to be implicated in the disease complex, including relatively novel pathogens such as canine influenza virus (CIV) [14], *Mycoplasma* sp. [15], *Streptococcus equi* subsp. *zooepidemicus* (*S. zooepidemicus*) [16], canine respiratory coronavirus (CRCoV) [8], and canine pneumovirus (CnPnV) [17]. Canine reovirus [18], canine bocavirus [19], and canine hepacivirus [20] have been recently identified in CIRDC, not as big factors as some of the other agents, but their causal role and contribution to the complex have not yet been elucidated.

The CIRDC pathogens are often highly infectious and transmitted mostly by an aerosol route, particularly when many dogs are housed in high-density environments such as boarding kennels, rehoming centers, and shelters [9,21]. Close contact, limited ventilation, incomplete vaccination, frequent turnover of dogs, and the stress associated with kenneling can all contribute to an increased risk of outbreaks of CIRDC in these settings. Further, outdoor dog parks, daycare facilities, and even veterinary clinics may present the same opportunities for crowded spaces, due to the large number of dogs, and can also potentially facilitate CIRDC transmission. For instance, precedent reports have described an outbreak associated with canine parainfluenza virus and canine herpesvirus among dogs in veterinary hospitals [4,22]. Because the clinical signs are not sufficient to distinguish between the various causes of CIRDC, laboratory diagnostic tests are crucial and are routinely performed for determining the etiologic agent. The commonly used laboratory diagnostic assays for the detection of pathogens associated with CIRDC include virus isolation, bacterial culture, enzyme linked immunosorbent assay (ELISA), polymerase chain reaction (PCR), and reverse transcriptase PCR (RT-PCR). Of the various laboratory diagnostic tests available, single plex and/or multiplex real-time PCR (rt-PCR) or real-time RT-PCR (rtRT-PCR) assays are currently the most commonly used tests as they are rapid, highly sensitive, and specific in detecting the various pathogens associated with CIRDC [23,24]. There is no specific treatment for the syndrome. However, it is recommended that antibiotics should be administered for primary and secondary bacterial infection [25,26]. Several monovalent and multivalent vaccines, some generally formulated for intranasal administration, are available for some pathogens but not for all, which may explain the occasional lack of protection. Another possible cause of that lack of protection could be justified by the presence of antigenic variants. Co-infection is frequent in CIRDC, and previous studies have shown that it may result in a complicated form of the disease, including pneumonia [6,7]. The role of viruses and bacteria as primary or secondary pathogens in CIRDC is still not fully understood.

The pathogenesis of CIRDC is a subject of ongoing research, and factors contributing to its complexity include co-infection dynamics, particularly understanding how co-infections occur and influence disease progression remains an active area of investigation. Additionally, individual variations in a dog’s immune system, age, and overall health can also impact how pathogens affect the respiratory tract. The role of host factors in the CIRDC pathogenesis is still not fully elucidated. Improving our understanding of how viral and bacterial agents interact, the influence of host and environmental factors, and the specific roles they play in the disease process will ultimately contribute to more effective diagnosis, prevention, and treatment strategies for CIRDC in dogs. Despite the availability of vaccines and antibiotic regimens adopted, outbreaks are still reported worldwide, and there is very little information available on the prevalence of CIRDC in the United States. The main objective of this study was to retrospectively conduct a data analysis on clinical samples submitted to a veterinary diagnostic laboratory for CIRDC testing to determine the prevalence of CIRDC pathogens over the 5 years from 2018 to 2022 and provide insights into the epidemiology of its etiologic agents.

## 2. Materials and Methods

### 2.1. Specimens

A total of 459 respiratory specimens (nasal, oropharyngeal, nasopharyngeal swabs, bronchoalveolar lavage fluid, etc.) collected over the five-year period from 2018 to 2022 were used in the study. The specimens were collected by veterinary practices across the southeastern United States from dogs showing respiratory signs and were submitted to the Athens Veterinary Diagnostic Laboratory (AVDL, University of Georgia, Athens, GA, USA) for CIRDC testing by PCR. The signalments accession number, received date, specimen types, age, and sex were recorded from the sample submission forms.

### 2.2. Nucleic Acid Extraction

Nucleic acids from the respiratory specimens were extracted by either IndiSpin Pathogen Kit (Indical Bioscience, Orlando, FL, USA) or IndiMag Pathogen Kit (Indical BioSciences), formerly MagAttract 96 cador Pathogen Kit (Qiagen, Germantown, MD, USA) according to manufacturer’s instructions. The extractions were performed using the QIAcube (Qiagen, Redwood City, CA, USA) or KingFisher Flex or KingFisher Apex purification systems (ThermoFisher Scientific, Waltham, MA, USA).

### 2.3. Polymerase Chain Reaction (PCR)

All PCR assays were performed with an exogenous internal control, which both ensured the quality of the extraction process and detected the presence of naturally occurring inhibitory compounds in the submitted specimens. The internal control was added into each sample during the extraction procedure to monitor the quality of both the purification and amplification. Either VetMax Xeno Internal Positive Control RNA (ThermoFisher Scientific) or VetMax Internal Positive Control—LIZ Assay (ThermoFisher Scientific) was used as internal control in each reaction. In addition, all quantitative real-time PCR assays were performed and analyzed following the guidelines as per the Minimums Information for publication of Quantitative real-time PCR Experiments (MIQE) [27,28].

The primers and probes used in the PCR assays are provided in Table 1. All PCR assays were performed and analyzed using standard procedures (please see references listed in Table 1). For CAV-2 detection from a respiratory specimen, a traditional gel-based PCR was performed using Fast-cycling PCR Master Mix (Qiagen) and the canine vaccine, Nobivac (Merck, Madison, NJ, USA) was used as a positive amplification control (PAC). In all PCR reactions, ultrapure nuclease-free water (ThermoFisher Scientific) was used as a negative control. A real-time RT-PCR was performed for the amplification of CDV using an Ag Path One-Step RT-PCR kit (ThermoFisher Scientific) and Nobivac vaccine as PAC. The CIV was amplified from clinical samples by a real-time RT-PCR using Ag Path ID (ThermoFisher Scientific) or the One-Step RT-PCR (Qiagen) run with AI matrix *203ADV0704* (National Veterinary Services Laboratories, USDA, Ames, IA, USA) as PAC. A nested PCR after reverse transcription (RT) of the extracted nucleic acid was performed for the amplification of CPIV. If the primary PCR was positive, no additional rest was run, but if the primary PCR was negative, the secondary nested PCR was followed before the results were reported [9]. The assay was run using One-Step RT-PCR kit (Qiagen) and CPIV *PUC57Kan* in *Escherichia coli* (developed in-house) as PAC. For CRCoV, a real-time RT-PCR was performed using Ag Path One-Step RT-PCR kit (ThermoFisher Scientific) with CRCoV *PUC57Kan* in *E. coli* (developed in-house) as PAC. For CHV-1 amplification, a real-time PCR using 2X QuantiFast Probe Master mix (Qiagen) was used along with a positive CHV-1 virus isolate (isolated and characterized in-house) as positive control. A real-time PCR amplification was performed for each one of the bacterial pathogens in the study, *B. bronchiseptica*, *S. zooepidemicus*, *M. canis*, and *M. cynos* using 2X QuantiFast Probe Master mix (Qiagen). The respective pure-culture bacterial isolates (isolated and characterized in-house) were used as PACs. Please see Table 1 for a list of references for detailed materials and methods, and for information on primers and probes.

### 2.4. Data Analysis and Statistics

We performed a retrospective analysis (2018–2022) by querying the database using the Laboratory Information Management System called VetView (Athens, GA, USA) to retrieve all submitted cases that requested a CIRDC PCR panel over the past 5 years (2018–2022). We split the seasonality into the cold (15 October to 15 April) and warm seasons (16 April to 14 October) to analyze the impact of seasons on the prevalence of CIRDC pathogens. To determine the rate of pathogen detection by age, we also divided the dog population into four categories: puppies (<1 year old), adolescents (1–4 years old) adults (4–10 years old), and seniors (>10 years old) dogs. Information about previous exposure, vaccination status, and treatment regimens was not assessed.

Data in this study were recorded in Excel spreadsheets and imported into the JMP software for statistical analysis (JMP Statistical Discovery, Cary, NC, USA). Associations between pathogen occurrence, sex, and seasonality variables were examined using Chi-square and Fisher’s test. Multivariable logistic regression was used to examine pathogen occurrence depending on the age variable. A *p*-value of <0.05 was considered significant.

No animal experiments were conducted as part of this study. Specimens collected and submitted by veterinarians from their canine patients were used for laboratory diagnostic testing and reporting of results back to the submitting veterinarian. All confidential animal and owner identification information were removed before compiling the data to maintain client confidentiality.

## 3. Results

**Figure 1:**
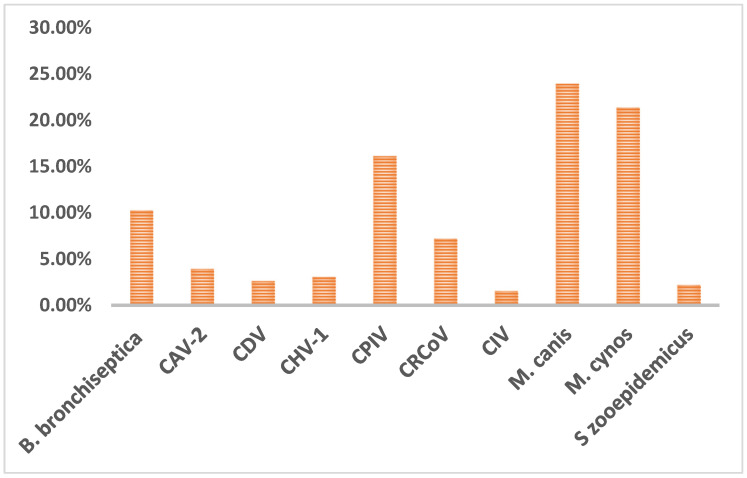
Overall prevalence of CIRDC pathogens.

The data were first summarized based on the results of bacterial and viral infections. Viral pathogens were detected in 34% (*n* = 158 out of 459 tested) of cases while bacterial infections were detected in 58% (*n* = 265) of the cases. Among the various pathogens tested, CPIV was the most predominant virus, and *M. canis* was the most predominant bacteria detected in CIRDC in dogs during the study period from 2018 to 2022. As illustrated in Figure 1, *M. canis* (24%, 110/459), *M. cynos* (21%, 98/459), and CPIV (16%, 74/459) were the most commonly detected pathogens, followed by *B. bronchiseptica* (10%, 47/459), CRCoV (7%, 33/459), CAV-2 (4%, 18/459), CDV (3%, 12/459), CHV (3%, 14/459), CIV (2%, 7/459) and *S. zooepidemicus* (2%, 10/459).

When single infection versus co-infection was examined, 31% of the cases were single infections with either one virus or one bacterium as the causative agent. Co-infection with more than one virus or bacteria was detected in 24% of the cases tested. Neither a virus nor bacteria were detected in 45% of the cases examined during the study period. Of the co-infections detected, co-infection with *M. canis* and *M. cynos* was the most frequent (*n* = 20) during the study period. Co-infection with CAV-2 and CPIV was detected in four cases, and co-infection with CPIV and *M. canis* was detected in nine cases. Other notable co-infections included: CPIV, *M. cynos*, and *B. bronchiseptica* (*n* = 3); CPIV, *M. canis*, and *B. bronchiseptica* (*n* = 3); CPIV, *M. canis*, and *M. cynos* (*n* = 3); and *M. canis*, *M. cynos*, and *B. bronchiseptica* (*n* = 3). Co-infections were detected in two cases each with CDV and CPIV, and CRCoV and CPIV, respectively. A co-infection involving four pathogens, CAV-2, CPIV, *M. canis*, and *M. cynos* was also detected in two cases.

**Figure 2:**
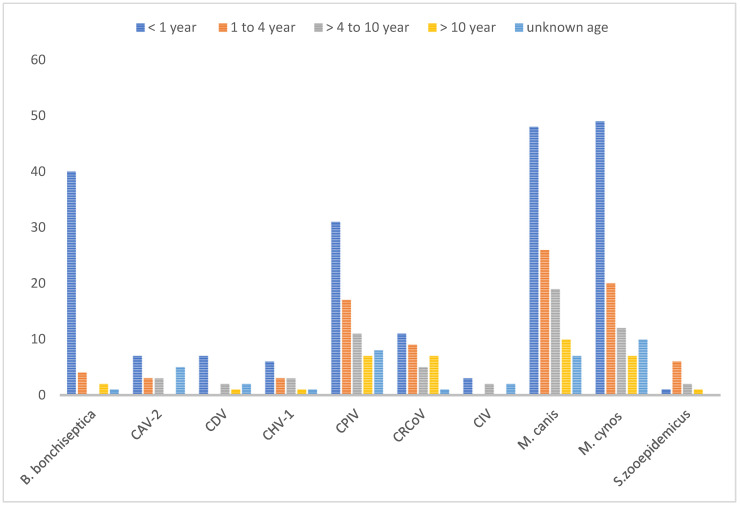
Age-wise distribution of CIRDC pathogens (2018–2022).

Regarding age susceptibility to infections, overall, dogs less than one year of age were the most susceptible population to infections by CIRDC pathogens. Some of the CIRDC pathogens were not detected in all age categories, namely *B. bronchiseptica* was absent in adult dogs, and CAV-2 and CIV were not detected in senior dogs. We also noticed the absence of CDV and CIV in adolescent dogs. Still, puppies were significantly more affected than other age groups with *B. bronchiseptica, M. canis* and *M. cynos*. (*p* < 0.0001, *p* < 0.04 and *p* < 0.0001 respectively). Adolescents were the second most affected age group and with the same pathogens as in puppies (Figure 2).

Out of 459 cases examined in the present study, 51% were male dogs, 44% were female dogs, and 6% were dogs with no documented details of gender identification. There was no significant relationship between the pathogen occurrence and the sex except for CDV and CIV. For CDV, positive cases were detected more often in males than in females (*p* < 0.0475) and for CIV, positive cases were detected more frequently in females than in males (*p* < 0.0054).

**Figure 3:**
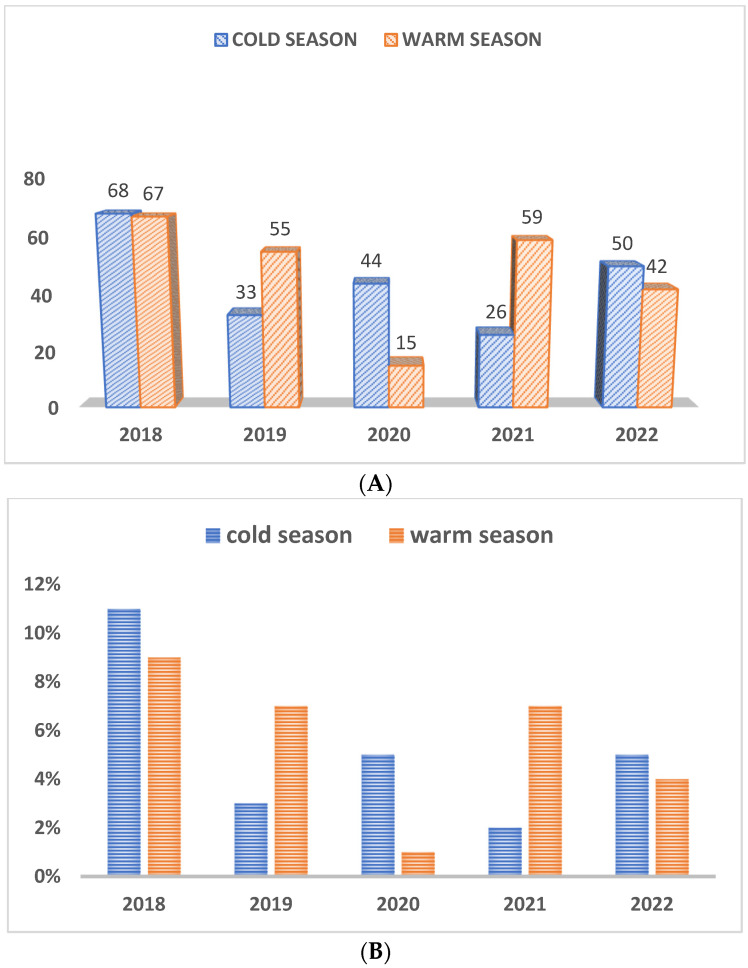
(**A**) Number of total cases (positive and negative) received during cold and warm seasons. (**B**) Prevalence of positives cases during cold and warm seasons as a percentage of total cases per year.

As depicted in Figure 3A, regarding the seasonality of infections, more samples were received during the warm weather (*n* = 238) than in the cold season (*n* = 221), but these case submissions fluctuated year by year, as shown in Figure 3A. Subsequently, the prevalence of positives cases also differed from year to year and did not show any particular trend between seasons (Figure 3B).

## 4. Discussion

The present study aimed to determine the prevalence of etiologic agents associated with CIRDC by doing a retrospective analysis of the cases submitted to the Athens Veterinary Diagnostic Laboratory, University of Georgia, Athens, GA. We explored many aspects of the CIRDC in the present study including the prevalence of pathogens by age, sex, and seasonality. Our findings showed that puppies were the most affected by the CIRDC and that pathogens such as CPIV, *M. cynos, M. canis* and *B. bronchiseptica* were predominantly associated with the disease condition. A previous study had found that younger dogs were also commonly infected, but with CRCoV instead, and they tended to develop more severe clinical signs [37]. The combination of an immature system, lack of prior exposure, increased stress, and socialization in environments with potential pathogen exposure may contribute to the common occurrence of CIRDC in younger dogs [37].

A previous investigation of the prevalence of Mycoplasma from tracheobronchial lavages and pharyngeal swabs had shown that dogs with and without pulmonary disease had Mycoplasma isolated from their pharynx [38], which revealed that Mycoplasma species may coexist in the respiratory tract of healthy dogs without causing illness, suggesting a commensal relationship. However, when the dog’s immune system is compromised or when other factors create conditions conducive to infection, *M. cynos and M. canis* become opportunistic and their relationship with dogs can switch from commensal to pathogenic. In many cases, viral infections were shown to play a significant role in compromising the integrity of the upper respiratory tract’s epithelium. Viruses such as CPIV often target and damage the respiratory epithelial cells, making the affected tissue more susceptible to secondary bacterial infections [39,40]. This sequential infection process can lead to increased destruction and inflammation of the upper respiratory tract. The initial viral infection weakens the defense mechanism of the respiratory tract, disrupts the mucociliary clearance system, and impairs the local immune response. This creates an environment where bacteria such as *B. bronchiseptica* or *Mycoplasma* sp. can more easily colonize and multiply [39,40].

A recent study [2] showed that asymptomatic healthy dogs can also act as a source of infection by CIRDC pathogens for susceptible dogs. Dogs housed in shelters were at a higher risk of asymptomatic carriage of pathogens associated with CIRDC compared to client-owned dogs, except for CHV-1. Between shelter and client-owned dogs, shelter dogs had a higher proportion of positive cases compared to client-owned dogs and a higher risk for the CIRDC pathogens examined, which included *M. cynos* (0.18, 95% confidence interval: 0.12 to 0.25), CRCoV (0.15, 95% confidence interval: 0.10 to 0.19), CDV (0.06, 95% confidence interval: 0.03 to 0.09), and canine pneumovirus (CnPnV) (0.05, 95% confidence interval: 0.03 to 0.08) [2]. In another study comparing the detection of CIRDC pathogens between clinically ill and healthy dogs, 37.7% dogs tested were positive for CPIV, 9.8% for CRCoV and 78.7% for *B. bronchiseptica* in clinically ill patients whereas 1.1% dogs were positive for CAV-2, 7.8% for CPIV and 45.6% for *B. bronchiseptica* in healthy dogs [39]. The same study showed that in dogs with CIRDC, co-infections with more than one viral or bacterial pathogen were detected in 47.9% of cases with *B. bronchiseptica* infections, 82.6% of cases with CPIV infections, and 100% of cases with CRCoV infections [39].

*M. cynos* was first isolated from kenneled dogs affected with pneumonia [41]. Since then, it has been primarily recognized for its ability to cause respiratory infections in dogs, and its implications in CIRDC continued to evolve since its initial discovery. The high prevalence of these Mycoplasma species in our study along with previous studies underscores their potential role in CIRDC. Another work identified *M. cynos* alone, and in association with other pathogens in symptomatic dogs, contributing to a spectrum of clinical signs ranging from mild to severe [6]. The results of a precedent study supported *M.* cynos as a primary agent playing a role in the lower respiratory tract while describing *M. canis* as commensal [42]. Overall, the presence of Mycoplasma, both as a sole pathogen and in combination with other infectious agents, poses a significant challenge in delineating its pathological role within CIRDC. Despite all, determining whether Mycoplasma functions as a primary initiator of disease or as a secondary opportunistic pathogen remains a complex task. Its ability to coexist with other pathogens complicates the attribution of causality and significance of Mycoplasma in the multifaceted landscape of CIRDC.

Canine parainfluenza virus is considered one of the main canine respiratory viral agents that belongs to the *Paramyxoviridae* family of RNA viruses, and was first isolated from laboratory dogs. It replicates in the lower and upper respiratory tract, causing mild lesions in the lungs [3], and was significantly associated with moderate to severe clinical signs [6]. Over time, we have seen fluctuations in the prevalence of CPIV in CIRDC. A higher prevalence among clinically ill than exposed and convalescent dogs has been previously reported [7]. Our data in addition to other reports [6,9] suggested that CIPV is an important CIRDC etiologic agent and that it remains in circulation despite the vaccination of dogs to protect against CPIV infection.

Kennel cough has always been seen as a regular common respiratory disease with truly little clinical significance that could be prevented through vaccination. In the past few years, the resurgence or emergence of new pathogens has progressively boosted the interest in this condition and is mostly seen now as a complex caused by multiple pathogens [43]. Some of those emerging pathogens, such as *S. zooepidemicus*, a commensal organism in horses, are often associated with opportunistic infections. *S. zooepidemicus* has been recently described as being involved in fatal hemorrhagic pneumonia in dogs in South Korea [44] and the United Kingdom [16]. *M. cynos*, was also isolated from several cases of lethal bronchopneumonia in puppies [45]. Additionally, CRCoV was shown to be prevalent and related to CIRDC complications in a European study [37]. Though not a part of the scope of the present study, CIRDC cases caused by CnPnV have also been reported from the United States and Europe for more than a decade [17,46]. Given these points, we could infer there might be a possibility that emerging pathogens are associated with the increasing severity of disease, which highlights the urge to implement good management and control measures. While *S. zooepidemicus* was not detected in another similar study [6], we identified some positive cases of *S. zooepidemicus* in the present study.

The present study also demonstrated an exceptionally low occurrence of traditional CIRDC pathogens, such as CDV, CAV-2 and *B. bronchiseptica*, while surprisingly noticing other pathogens such as *M. canis, M. cynos* being commonly identified. These results might be suggesting that the widespread use of vaccines in the United States is successful. Paradoxically, this could convey the need to implement new vaccine regimens that include emerging pathogens. The kennel cough multivalent vaccines have been developed conferring protection against CDV, CAV type 1 and 2 and CPIV, also available in combination with *B. bronchiseptica* but not with emerging pathogens such as *M. cynos*, and *M. canis.* Vaccination plays a significant role in CIRDC management by reducing the spread of pathogens and limiting the risk of developing a severe form of disease. Therefore, having more inclusive vaccines could be crucial to prevent the pathology. On the other hand, it has also been reported that, though vaccination would reduce the infection rates, duration of shedding and severity of disease, it might not confer sterilizing immunity [47]. This factor also needs to be considered while designing strategies for management, control, and prevention of CIRDC pathogens.

It is noteworthy that CIRDC is a constantly evolving syndrome with pathogens continuing to emerge, and new agents are being identified at an increasingly rapid rate, especially with the advance in sophisticated diagnostic techniques. Continued monitoring of CIRDC pathogens through rapid and accurate laboratory diagnostic testing and finetuning of vaccine strategies are critical for the prevention and control of CIRDC in dogs.

## Figures and Tables

**Table 1:** Primers and probes used in the polymerase chain reaction assays for the detection of canine infectious respiratory disease pathogens in respiratory specimens in dogs. * In-house probe.

Pathogen	Test Method	Primers/Probes	Reference
Canine adenovirus type 2 (CAV-2)	Traditional/Gel-based PCR	CAV-F: 5′-CGC GCT GAA CAT TAC TAC CTT GTC-3′ CAV-R: 5′-CCT AGA GCA CTT CGT GTC CGC TT-3′	[29]
Canine Distemper virus (CDV)	Real-time RT-PCR	CDV-F: 5′-ACT ATT GAG AGA CCT CCA GCT GAA A-3′ CDV-R: 5′-TGC GGT ATC CTT CGG TTT GT-3′ CDV-P: 5′-/6-FAM/CCG ATT GCC GAG CTA GAC TCT TTG TCA/BHQ-1/-3′	[30]
Canine influenza virus (CIV)	Real-time RT-PCR	M + 25 F: 5′-AGA TGA GTC TTC TAA CCG AGG TCG-3′ M-124-2002 R: 5′-TGC AAA AAC ATC TTC AAG TCT CTG-3′ M-124-2009 R: 5′-TGC AAA GAC ACT TTC CAG TCT CTG-3′ M+ 64 P: 5′-/6FAM/TCA GGC CCC CTC AAA GCC GA/3IABkFQ/-3′	[31]
Canine Parainfluenza virus (CPIV)	Nested PCR	Primary PCR PNP1 F: 5′-AGT TTG GGC AAT TTT TCG TCC-3′ PNP2 R: 5′-TGC AGG AGA TAT CTC GGG TTG-3′ DNA standard Secondary PCR PNP3 F: 5′-CGT GGA GAG ATC AAT GCC TAT GC-3′ PNP4 R: 5′-GCA GTC ATG CAC TTG CAA GTC ACT A-3′	[9]
Canine Respiratory Coronavirus (CRCoV)	Real-time RT-PCR	CRCoV-F: 5′-ACG TGG TGT TCC TGT TGT TAT AGG-3′ CRCoV-R: 5′-AAC ATC TTT AAT AAG GCG ACG TAA CAT-3′ CRCoV-P: 5′-/6-FAM/CCA CTA AAT TTT ATG GCG GCT GGG ATG/3IABkFQ/-3′	[32]
Canine herpesvirus type 1 (CHV-1)	Real-time PCR	CHV-F: 5′-ACA GAG TTG ATT GAT AGA AGA GGT ATG-3′ CHV-R: 5′-CTG GTG TAT TAA ACT TTG AAG GCT TTA-3′ CHV-P: 5′-/6-FAM/TCT CTG GGG TCT TCA TCC TTA TCA AAT GCG/BHQ-1/-3′	[33]
*Bordetella bronchiseptica*	Real-time PCR	bfr-Q F: 5′-CGGAGTGAGATCGTGCATCA-3′ bfr-Q R: 5′-CCACCAAACGCAATGACCTG-3′ bfr-P: 5′-/6FAM/TCGGGAAGGTGCAGCATGTCCTGGAAATA/BHQ-1/-3′	[34,35]
*Streptococcus zooepidemicus*	Real-time PCR	SodA-F: 5′-AGA GCA ATT CAC AGC AGC A-3′ SodA-R: 5′-ACC AGC CTT ATT CAC AAC CA-3′ SodA-Bd-R: 5′-ACC GGC TTG GTT AAC CAC TA-3′ SodA-P: 5′-/6-FAM/CAG GCC CAA CCT GAG CCA AA/36-TAMSp/-3′	[36]
*Mycoplasma canis*	Real-time PCR	F: 5′-CAC CGC CCG TCA CAC CA-3′ R: 5′-CTGTCGGGGTTATCTCGAC-3′ P*: 5′-/6FAM/TTATCAATTATTATTTTAAATGTCA /3MGBEc/-3′	[15]
*Mycoplasma cynos*	Real-time PCR	F: 5′-CAC CGC CCG TCA CAC CA-3′ R: 5′-GATACATAAACACAACATTATAATATTG-3′ P *: 5′-/5JOE/3NHS/CGGAGTACAAGTTACAATTCATTTTAG/3IBFQ/-3′	[15]

## Data Availability

No additional data is available due to privacy or client confidentiality policy of the Athens Veterinary Diagnostic Laboratory.
